# Treatments for ADHD in adults in jails, prisons and correctional settings: a scoping review of the literature

**DOI:** 10.1186/s40352-023-00234-9

**Published:** 2023-09-07

**Authors:** Cory Byrne, Dale Guenter

**Affiliations:** 1 School of Interdisciplinary Science, Faculty of Science, McMaster University, 1280 Main St W, Hamilton, ON L8S 4L8 Canada; 2 Department of Family Medicine, Faculty of Health Sciences, McMaster University, 100 Main St W, Hamilton, ON L8P 1H6 Canada

**Keywords:** Attention-deficit/hyperactivity disorder (ADHD), Treatment, Intervention, Stimulants, Inmates (people who are incarcerated (PWAI)), Correctional setting, Prison, Review

## Abstract

**Background:**

Attention-Deficit / Hyperactivity Disorder (ADHD) is prevalent at a higher rate in correctional settings than in the general population. Treatment of ADHD in this environment is challenging as stimulants, the most common treatment for ADHD, require cautious prescribing in the context of frequent substance use disorders (SUD) and diversion in the institutional setting. In addition, both pharmacological and non-pharmacological treatment approaches require significant staff resources. The aim of this scoping review is to map and summarize all literature addressing treatment of ADHD specifically in correctional settings, synthesize the evidence supporting various approaches, and highlight areas for future research. Due to the limited number of primary research studies addressing this question directly, we chose a scoping review methodology that would allow us to explore what kinds of studies and literature exist and include all types of articles directly related to our question.

**Results:**

Five-hundred sixty-five relevant articles were screened. Thirty-two articles were included in the final review. Eleven of the articles reported primary research. Five of these articles were based on three randomized controlled trials. Among randomized controlled trials on the effect of stimulants, inclusion criteria, outcome measures and effectiveness varied widely. Non-male and non-white populations were under-represented. Among review articles and recommendations opinion was inconsistent, with some recommending stimulants as first-line treatment and others recommending they be avoided altogether. The effect of non-stimulant medications was examined in 2 small studies. The only non-pharmacological treatment examined was dialectical behavior therapy, and only feasibility was reported. Four articles provided practice recommendations from consensus or expert opinion. Two of these recommended stimulants as first-line treatment, and two recommended stimulants as treatment of last resort.

**Conclusions:**

We found a diverse but shallow literature addressing our research question. Primary research in the corrections setting is limited and varies in inclusion criteria, outcomes studied, and effectiveness. Recommendations on treatment are inconsistent. Future research should address methods of diagnosis, the role of non-stimulants, non-pharmacological interventions, non-male and non-white people who are incarcerated (PWAI), and effects of treatment method on patients, staff and other PWAI. Better research and guidance on treating ADHD has potential to improve health of PWAI, the institutional environment, and resource utilization.

## Background

### Definition of attention-deficit/hyperactivity disorder

Attention-deficit/hyperactivity disorder (ADHD) is a neurodevelopmental condition characterized by inattention, hyperactivity, or impulsivity that interferes with daily functioning, interpersonal activity, and academic achievement. Although most commonly diagnosed in childhood, ADHD can continue into adulthood. Diagnosis of ADHD is extremely challenging and subjective. As of 2013, the major method used by practitioners to diagnose ADHD is based on DSM-5 criteria. For adults 17 years and older, diagnosis requires at least 5 symptoms of either inattention or hyperactivity lasting at least 6 months. In addition, the symptoms must have been present since before age 12, present in at least two different settings, not caused by other disorders, and interfere with the individual’s quality of life (American Psychiatric Association, 2013). Examples of inattention symptoms include trouble maintaining attention, forgetfulness, and failure to pay close attention to details. Examples of hyperactivity symptoms include fidgeting, excessive talking, and an inability to stay seated for long periods of time. Other methods for diagnosing ADHD include various ADHD self- or clinician-rated screening tools. Each tool is subjective and unique, which creates inconsistencies with ADHD diagnosis. Overall, diagnosing ADHD is difficult due to the lack of objective criteria, symptomatic behaviours being on a spectrum with normal behaviour, and the possibility of other comorbid mental health conditions causing similar symptoms (Katzman et al., 2017).

### ADHD prevalence in correctional facilities

The Canadian Community Health Survey estimated the prevalence of clinician-diagnosed adult ADHD within the general population to be 2.7% (Connolly et al., 2019). The prevalence of ADHD has consistently been found to be higher in correctional settings than in the general population. Diverse studies estimate the prevalence of ADHD in correctional facilities in the range from 9.1 to 45% (Beaudry et al., 2021; Billstedt et al., 2017; Blocher et al., 2001; Cahill et al., 2012; Curran & Fitzgerald, 1999; Eyestone & Howell, 1994; Ginsberg et al., 2010; Hamzeloo et al., 2016; Konstenius et al., 2015; Lindgren et al., 2002; Moore et al., 2016; Rösler et al., 2004). A systematic review of 102 studies and 69,997 participants from detention settings, 27.5% of which were adults, reported an adult ADHD prevalence of 26.2% (Baggio et al., 2018). The review found no significant difference between prevalence rates identified using screening tools or clinical interviews. Specifically in Canada, one study found the prevalence of adult ADHD in 497 Canadian males experiencing incarceration to be 16.5% based on the Adult ADHD Self-Reporting Scale (ASRS) (Usher et al., 2013).

Behaviours concurrent with ADHD such as increased defiant and antisocial behaviour, and substance use disorders (SUD), are likely to play a role in the observed higher prevalence in correctional facilities (Ginsberg et al., 2010). A study by Velez-Pastrana et al. (2020), found individuals experiencing incarceration with ADHD had a significantly higher risk for lifetime SUD (OR = 2.17) and current SUD (OR = 2.08).

### ADHD treatment in adults

Much of the clinical guidance for treating adults in the general population who suffer from ADHD recommends simulants as the first line pharmaceutical approach. However, there remains significant uncertainty about the value of, and best approach to, treatment of ADHD in adults (Huang et al., 2020; Castells et al., 2018; Cândido et al., 2021; NICE, 2018; CADDRA, 2020). This uncertainty arises from challenges with diagnosis (especially in the context of co-morbidity with other mental illness and addiction), heterogeneity and low quality of scientific methods, inconsistency in the clinical outcomes that show improvement, and a short duration of follow-up for most studies. In addition, treatment with stimulants is difficult as they may be abused and lead to negative health outcomes. Stimulants can also cause safety concerns in correctional facilities due to diversion. Although non-stimulants can be used with less risk of abuse, they do not have the same short-term effectiveness as stimulants (Cortese et al., 2018). And finally, a recent Cochrane review of immediate-release methylphenidate for adult ADHD in general populations concluded there was no certain evidence that it could reduce ADHD symptoms more than lithium or placebo treatment (Cândido et al., 2021).

Due to constraints of time and resources, and the movement of individuals between institutions, nonpharmacologic treatments may be challenging to provide in correctional settings (Young & Cocallis, 2019). Furthermore, screening for ADHD within these settings is often inadequate, preventing individuals from being identified for treatment. These diagnostic and treatment challenges combined with ADHD’s high prevalence in correctional facilities pose a complex challenge.

### Study purpose

There is currently no widely agreed upon treatment method for individuals with ADHD in correctional facilities. While there is an abundance of literature relating ADHD to criminality or addressing treatments outside of correctional settings, this review will focus solely on treatments within institutions, for which the body of literature is much smaller. A scoping review was chosen for our inquiry due to the diversity of literature currently available. Our study goals are to map the types of articles and research studies, describe the range of research methods and outcomes, identify areas of consensus and controversy, identify research gaps, and ultimately inform treatment recommendations and guidelines.

## Methods

The methodology for our scoping review follows the 5-step approach outlined by Arksey and O’Malley (2005): (1) identifying the research question, (2) identifying relevant studies, (3) study selection, (4) charting the data, and (5) collating, summarizing and reporting the results. In keeping with scoping review methodology, our research question evolved somewhat as study questions and methods included in the literature became evident. A search strategy was developed in consultation with a university librarian. The initial literature search was conducted in October 2021 and updated in December 2022. It included the search terms identified in Table 1. The final research question was: what treatments have been studied, showing what effectiveness, among people in correctional facilities?

**Table 1:** Key terms used in the literature search

Attention Deficit Disorder with Hyperactivity AND	Correctional Facilities AND	Therapeutics
Attention deficit hyperactivity disorder OR attention deficit disorder with hyperactivity OR attention deficit disorder* OR attention deficit hyperactivity disorder* OR attention deficit-hyperactivity disorder* OR ADHD OR ADDH	Prison* OR jail* OR correction* facilit* OR detention centre* OR detention center* OR incarcerated OR detainee* OR remand OR confinement	Pharmac* OR drug* OR intervention* OR health care OR therapeutic* OR therapies OR therapy OR treatment*

**Fig. 1:**
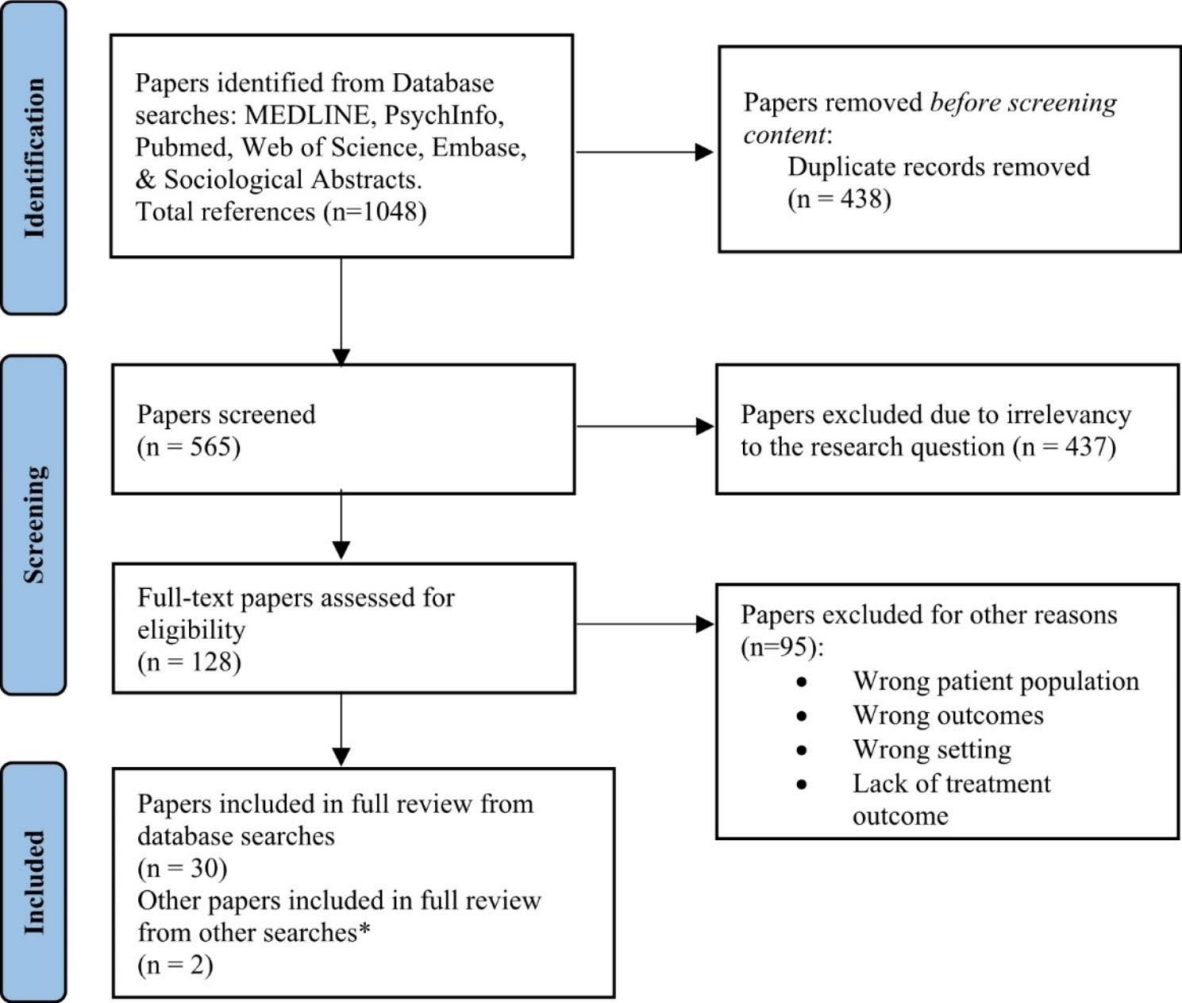
PRISMA diagram of selection process for articles (Page et al., 2021) * *Both articles were published after the date of the database search and were found by authors when reading literature relating to the scoping review*

The search was conducted in the following databases: Ovid MEDLINE Epub ahead of print (1946–2021), APA PsychInfo, Pubmed, Web of Science, Embase, and Sociological Abstracts. We did not conduct a search of the grey literature. Relevant studies were identified as those that involved the following: (a) adult populations in correctional settings and (b) the treatment of (c) ADHD. All types of research papers from any year were included. Articles were reviewed using the Covidence web platform. Both researchers screened the titles and abstracts of the 565 articles resulting from the initial search to determine inclusion for full review. Inclusion criteria for this step were kept broad to identify as many relevant articles as possible. Of these articles, 128 were selected and underwent a full-text review by one researcher (CB) to further determine relevance to the research question. Thirty of these articles fit the scope of our research goals and an additional 2 relevant articles that were published after the date of the initial search were included in the final report. Figure 1 shows a PRISMA diagram of the complete process.

## Results

### Mapping the characteristics of articles reviewed

The outcome of our final review is summarized in Tables 2, 3 and 4. Articles are mapped as types of studies by country of origin in Table 2. Methods and results of each primary research study are summarized in Table 3, and all other studies in Table 4.

**Table 2:** Types of articles by country of origin

Article Type	Sweden (n = 6)	USA (n = 11)	UK (n = 8)	Spain (n = 1)	Switzerland (n = 1)	Germany (n = 1)	Canada (n = 2)	Multi-national (n = 2)
**Primary Research (n = 11)**	5	4	2	0	0	0	0	0
RCT* Quasi-experimental Case series Chart review Evaluation/QI**	4 1 0 0 0	0 1 1 1 1	1 0 0 0 1	n/a	n/a	n/a	n/a	n/a
**All Other Articles (n = 21)**								
Non-systematic review, consensus statement, expert opinion, recommendation	1	7	6	1	1	1	2	2

*Notes:*

*Two separate randomized controlled trials (RCTs) were completed. One RCT had one article published and the other had three separate papers published, thus totaling four RCT articles

**Evaluation of implementation of program or approach, or strategy to improve quality (QI)

**Table 3:** Summary of primary research articles

Author/Year	Study Type	Country	Intervention	Population	Screening/Diagnosis	n	Follow-up (Weeks)	Results	COI*
Asherson 2022	RCT	UK	OROS-MPH	Male 16–25	Barkley/clinical interview	200	8	No difference in ADHD symptoms or secondary outcomes self and observer rated.	Y
Ginsberg, 2012	RCT	Sweden	OROS-MPH	Male 21–61	WURS-25/ASRS/clinical interview	30	5 + 47 open label ext.	Treatment improved self- and observer-rated ADHD symptoms. Treatment and placebo both improved during open-label extension. No drug misuse detected.	Y
Ginsberg, 2012	RCT	Sweden	OROS-MPH	Male 21–61	WURS-25/ASRS/clinical interview	30	5 + 47 open label ext.	Treatment improved verbal working memory, visuospatial working memory, verbal abstract reasoning, motor activity, and some aspects of quality of life.	Y
Ginsberg 2015	RCT	Sweden	OROS-MPH	Male 21–61	WURS-25/ASRS/clinical interview	24 (year 1), 20 (year 3)	5 + 47 open label ext. Follow up at 1 & 3 year	ADHD symptom improvement maintained at 1- and 3- year follow up. Participants continuing medication had less ADHD symptoms, alcohol/drug misuse, and functional impairment.	Y
Konstenius 2014	RCT double blind	Sweden	OROS-MPH	Male 18–65 w/ amphetamine dependence; final 2 weeks before release	WURS/ASRS/CCPT/clinical interview	54	24	Compared to placebo, treatment group improved self- and clinician-rated severity, sig more drug-negative urines, longer median retention to treatment, longer time till relapse, and decreased cravings compared to placebo.	N
Jillani 2016	Case series	USA	Atomoxetine	Male 16–20	Clinical interview	5	10	Investigator-rated ADHD symptoms improved and reduction in anxiety symptoms.	Y
Bastiaens 2019	Chart review	USA	Non-stimulants	PWAI with ADHD	Clinical interview	108	n/a	ADHD symptoms improved on average. Patients with no history of stimulant use had better treatment response.	N
Muld 2016	Quasi-experimental	Sweden	DBT	Males in compulsory care	WURS / ASRS / Clinical interview	40	6	Improved Self rated ADHD symptoms and general well-being, but no change in staff ratings.	N
Chaplin, 2021	QI	UK	Improvements to ADHD pathway	Males	brief-BAARS / DIVA-5	n/a	n/a	Structured screening, staff training, and other improvements lead to more ADHD diagnosis and treatment.	NS
Martin, 2006	Quasi-experimental	USA	EEG biofeedback	Male 14–17	ADHD diagnosis or psychologist screen	7	7–10	ADHD symptoms improved, worsened, or didn’t change for different participants. Some improvements in secondary measures.	NS
Appelbaum, 2011	Evaluation	USA	Treatment program pathway	Male	n/a	16,795	n/a	Over 2 years, 116 received stimulant treatment out of 16,795 candidates. 11 had treatment withdrawn due to misuse.	N

Notes: *COI (Conflict of Interest): Y = present, N = not present, NS = not specified

**Table 4:** Summary of review, recommendation and opinion articles

Author/Year	Article Type	Country	Main Points	COI
Young 2018	Consensus	UK	Screen all PWAI for ADHD. Stimulants first line, non-stimulants when ineffective/SUD. Include psychoeducation, psychological, and psychosocial components. Treat severe comorbidity before ADHD. Promote significant PWAI engagement in treatment.	Y
Appelbaum, 2009	Protocol	USA	Diagnosis: Consensus by 2 psychiatrists, diagnosis of ADHD before age 12, psych testing by doctoral psychologist, and clinically significant behaviour impairment. Treatment: Non-pharm first line, non-stimulants second line, stimulants if others not effective. Continuation of treatment requires objective improvement and compliance	NS
Scott 2016	Expert opinion	Canada	Modification of Appelbaum, 2009. Suggests addition of improved screening and behavioural interventions. First line treatment should depend on facility.	Y
Young, 2011	Consensus	UK	Suggests improved screening, staff training, and resource availability to PWAI. Methylphenidate first-line, atomoxetine/dexamphetamine when abuse potential. Treat comorbidities. Benefits of stimulants outweigh risks.	Y
Mattes, 2016	Review	USA	Stimulant risks may outweigh benefits. Mixed research on current medications. Alpha-2 agonists potential medication.	N
Knecht 2015	Systematic review	Spain	Self-report tools debatable, Coolidge Correctional Inventory specifically made for PWAI. Medication first-line when less severe symptoms. Tailored interventions needed if comorbidities.	Y
Young, 2019	Review	UK	Some concerns of stimulants may be unsubstantiated. Non-stimulants can be used when comorbid SUD. Focus on psychological, behavioural, and educational needs.	Y
Sebastian 2019	Review	Germany	Focus on substance use interventions and reduce violence/offending. Completion/adherence to rehabilitation plans is likely challenge. Early intervention programs required.	Y
Tully, 2022	Review	UK	Proper diagnosis/treatment of ADHD time-consuming, takes away from treating other conditions. Current research on medications is limited & questionable. Be cautious when prescribing.	N
Retz 2021	Review	Multi-national	Early recognition and intervention essential. Concerns of stimulants may be outweighed by benefits. Atomoxetine good alternative when comorbid SUD. Address psychological, behavioral, and educational needs.	NS
Sutton, 2016	Text & opi-nion	Canada	Non-stimulants may not be ideal in prison. Continuous performance tests may reduce malingering. Calls for practice guidelines, currently lack of consistency.	N
Appelbaum, 2008	Review	USA	PWAI staging behaviour can overburden psychiatrists. Guidelines/ consistency may mitigate issues. Misuse/diversion should prompt discontinuation of stimulants. Education/group therapy may benefit PWAI.	N
Burns, 2009	Text & opi-nion	USA	Discourages use of stimulants due to prevalence of SUD, misuse potential, and burden/safety concerns.	NS
Young, 2011	Review	Multi-national	Treatment requires complex plan that considers rehabilitation/comorbidity consideration. Early intervention crucial.	N
Ginsberg 2013	Text & opi-nion	Sweden	Medication can be beneficial but should be used carefully. Pros outweigh cons if treatment controlled and individualized.	Y
Hall, 2016	Commentary	USA	Diagnosis made difficult by high rates of trauma, SUD, and comorbidity. Treatment should include behavioral component.	N
UKAAN, 2013	Chapter in book	UK	Comprehensive, individualized treatment programs needed.	NS
Barry, 2008	Review	USA	Consider biological/genetic components in treatment.	NS
Young, 2015	Conference abstract	UK	Multimodal treatments may have a greater effect. R&R2ADHD developed for corrections.	Y
Fructuoso, 2019	Letter to editor	Switzerland	Stimulants are effective treatment, but major risk of misuse/diversion/safety for PWAI and staff. Suggests consideration of alternative approaches, such as non-addictive drugs or non-pharm interventions.	NS
Boutwell 2020	Review	USA	Stimulants are effective but concerned about safety/cost/side effects.	NS

### Summary of themes arising

#### Diagnosis and screening

Success of treatment will inevitably depend on the reliability of the diagnostic method. We found diverse approaches to diagnosis in the primary research studies reported. Among these studies, 6 used a clinical interview, 4 used the ASRS, 3 used WURS, 1 used CCPT, 1 used the brief-BAARS, 1 used the self-rated Barkley ADHD scale, and 3 were unclear/unspecified regarding what diagnosis or screening method was used. The UK expert consensus statement suggests the specific use of CHAT in youth followed by SNAP-IV / Conners’ CBRS, and the brief-BAARS in adults followed by an interview with CAADID, DIVA-2, or ACE+ (Young et al., 2018).

Authors discussed this inconsistency as one of the largest barriers to treating ADHD in corrections, along with the common presence of co-morbid mental health, addiction and personality disorders. Some suggest the creation of a standardized approach to screening and diagnosis of ADHD (Young et al., 2011, 2018; Scott et al., 2016; Knecht et al., 2015; Sutton & Kolla, 2016). Such guidelines could also be used to reduce burden on correctional clinicians (Sutton & Kolla, 2016). In addition to this, some articles suggest systematic ADHD screening for PWAI, either upon entry to any institution or on a regular basis (Young et al., 2011, 2018).

#### Pharmaceutical treatment

Most primary studies that we identified tested the effects of stimulants. Among the RCTs looking at methylphenidate, one found significantly improved observer-rated and self-rated ADHD symptoms, including clinician-rated severity and global functioning, cognitive measures, motor activity, and quality of life, compared to placebo (Ginsberg & Lindefors, 2012; Ginsberg et al., 2012). Observer and self-reported ADHD symptoms remained improved in 1- and 3-year follow up (Ginsberg et al., 2015). Another RCT found methylphenidate significantly improved self-reported ADHD symptoms and clinician-rated severity, but not clinician-rated improvement, compared to placebo (Konstenius et al., 2014). This RCT also found the treatment group had significantly less drug-positive urine tests. The most recent and largest RCT of methylphenidate found no significant differences in any measured outcome between treatment and placebo groups (Asherson et al., 2022). Additionally, more adverse events occurred in treatment groups than in placebo groups for all 3 RCTs.

Primary studies of non-stimulant treatment of ADHD in correctional settings were limited. One case series of 5 individuals on atomoxetine found it was able to reduce investigator-rated ADHD symptomatology and anxiety (Jillani et al., 2016). A retrospective chart review of atomoxetine and alpha-2 agonists showed that both improved clinician-rated severity (Bastiaens et al., 2019). In addition to primary research studies, one review was focused on the potential of alpha-2 agonists and suggested them as a potential treatment for patients in corrections facilities with ADHD (Mattes, 2016). The majority of review and opinion articles were in support of the use of medication for ADHD treatment. Most authors view stimulant medication as a component of multimodal treatment that would reduce symptomology to allow patients to gain optimal benefit from non-medical treatments (Ginsberg et al., 2013).

#### Non-pharmacological interventions

Non-pharmacological interventions are a component of multimodal treatment. Although we found only limited research evidence, there is significant expert support for non-medical interventions as effective treatments. These interventions are challenging to implement in correctional facilities due to considerations such as restricted resources. PWAI also have unpredictable time in institutions due to transfers or releases (Young & Cocallis, 2019), making shorter programs preferrable (Young et al., 2018). Only 2 primary studies utilizing non-pharmaceutical treatment were found. EEG biofeedback had mixed results on ADHD symptoms and cognitive measures, which were only completed on a small sample of patients (Martin & Johnson, 2006). DBT was found to be a feasible treatment option that significantly reduced self-rated ADHD symptoms (Muld et al., 2016). Overall, authors generally suggested key components of treatment should include education about ADHD, psychotherapy, behavioural control, and mentorship (Young et al., 2018; Scott et al., 2016; Appelbaum, 2008; Fructuoso, 2019). The R&R2-ADHD treatment program is a brief group cognitive skills program that has been widely promoted in publications by its creator, Susan Young. This program received support in 11 articles, 6 of those co-authored by the program creator. Primary studies of this treatment among people experiencing incarceration were not published by the time we conducted our review (Young & Ross, 2021). Finally, several articles recommended frameworks that should be adopted when treating ADHD in corrections. The Risk-Needs-Responsivity model was mentioned in 3 articles and ensures PWAI are given an appropriate service that matches their risk of recidivism (Young et al., 2011; Sebastian et al., 2019; Sutton & Kolla, 2016).

#### Impacts on the institutional environment

There were no measures of impact on the institutional environment of the corrections facilities, other PWAI, clinicians, or other correctional staff. However, review articles have speculated that treatment is likely to reduce problematic behaviours, aggression, and potentially comorbidities (Mattes, 2016; Muld et al., 2016). Improved screening may also be validating for PWAI (Tully, 2022). One author speculated that if PWAI believe they can easily acquire prescriptions for stimulants, it may overburden psychiatrists and have effects on the correctional environment (Appelbaum, 2008).

#### Controversy surrounding the use of stimulants

Stimulants are, on one hand, the most studied treatment for ADHD both in the general population and, as we have observed in our study, in people within correctional settings. On the other hand, their potential to cause physical dependency and harmful side effects is reported as being considerable. As described above, primary studies focusing on the effectiveness of stimulants in correctional settings have shown conflicting results. It is not surprising then that we identified review, opinion and consensus articles offering conflicting advice about ADHD treatment and policy.

Several concerns are raised. Due to the high comorbidity with substance use disorders the potential for misuse and diversion is raised frequently (Scott et al., 2016; Young et al., 2011; Mattes, 2016; Young & Cocallis, 2019; Sebastian et al., 2019; Knecht et al., 2015; Appelbaum, 2008; Burns, 2009; Ginsberg et al., 2013; Fructuoso, 2019). In addition, some authors raise concern regarding risk of burden on limited staff resources with the increased workload in storing and monitoring administration of stimulants, as well as risk to the security of both PWAI and staff (Young & Cocallis, 2019; Appelbaum, 2008; Burns, 2009). We did not identify empirical evidence to support these concerns. Additionally, there are concerns about the potential side effects of stimulants (Boutwell et al., 2020). We found empirical evidence for side effects among the primary studies that utilized stimulants, most of which were reported as mild to moderate (Ginsberg & Lindefors, 2012; Konstenius et al., 2014). Following from all of these concerns, some authors conclude that risk may outweigh benefit in the prescribing of stimulants in correctional settings (Mattes, 2016; Burns, 2009; Boutwell et al., 2020), while others support the use of stimulants as first line treatment especially when extended release and water soluble formulations are used, decreasing risk of diversion (Young et al., 2018; Scott et al., 2016; Young & Cocallis, 2019; Knecht et al., 2015; Retz et al., 2021; Sutton & Kolla, 2016). Protocols used to manage other kinds of controlled medications (Young et al., 2011, 2018; Scott et al., 2016; Young & Cocallis, 2019), and use of continuous performance tests to monitor for diversion (Sutton & Kolla, 2016) are examples of approaches suggested for decreasing misuse and diversion. Still, we did not identify empirical evidence concerning the benefit of these procedures from articles in our scoping review.

Some authors suggest the abuse potential of stimulants is overstated as a link between stimulant use and subsequent abuse has not been established (Scott et al., 2016). Some cite that research has found stimulant use in general populations can prevent SUDs if treatment predates the SUD and is well supervised (Young & Cocallis, 2019; Hall et al., 2016). It has been suggested that it is unethical to withhold stimulants from PWAI, since they are an accepted standard of treatment in the general population (Young & Cocallis, 2019). It has also been suggested that it is unethical to provide stimulant treatment to PWAI with the current evidence available (Tully, 2022). Some authors support stimulants as first-line treatments, but recommend using non-stimulants when SUDs are present (Young et al., 2011, 2018; Young & Cocallis, 2019), while others argue that the high prevalence of SUDs in PWAI is reason enough to avoid the use of stimulants altogether (Burns, 2009).

#### Gaps in research knowledge

There are significant gaps in what we found in primary, empirical research regarding treatment of people with ADHD in correctional facilities. First, there is little demographic diversity, with only one primary study including women. Only three primary articles mentioned racial demographics. All three studies were conducted on predominately white identifying individuals (62.5%, 90%, 82%), highlighting a lack of racial diversity in the current literature (Asherson et al., 2022; Bastiaens et al., 2019; Chaplin et al., 2021). There were only 2 studies of non-stimulant medications, both with no control group and with small samples. Studies of non-pharmacological treatment were few, small and also not controlled. There is a gap in consensus-based, consistent screening and diagnosis, and outcome measures. There is no research on the impact of treatment on the working and living environment of correctional facilities. And finally, we found no primary research addressing the impact of ADHD treatment in correctional facilities on relevant outcomes in the community following release such as reduced SUD, recidivism, and improved quality of life.

## Discussion

We found that the scientific knowledge base related to ADHD treatment in correctional settings has only a small number of primary studies. These studies are made up of small samples that are overwhelmingly white males, primarily focused on stimulants as treatment, diverse in methodology and outcomes, short in follow-up and inconsistent in results. We identified articles offering narrative review, expert opinion, and recommendations. These vary widely in their guidance. This presents a challenging foundation on which to base policy and clinical practice guidance for clinicians caring for people in correctional facilities.

Although not the subject of this scoping review, there is a substantial knowledge base that draws on primary research on ADHD treatment outside of the correctional facility context. This evidence is relevant to the treatment of ADHD in PWAI, but the degree to which it can be extrapolated is uncertain. People in correctional facilities have higher rates of comorbidity with SUD and mental illness than people with ADHD in the general population. In addition, patterns of socialization, interaction with facility staff and policies, prevalence of violence, and drug market dynamics within institutions will all play a role in both the need for, and the effectiveness of, ADHD treatment.

The unique and complex factors surrounding ADHD diagnosis and treatment in correctional facilities require specific and relevant treatment research to inform policy and clinical practice. We suggest that the current knowledge base is not adequate for this purpose. Our study sheds light on the critical need for research conducted among PWAI, as well as clinicians and institution staff. This research needs to be guided by stakeholder input including people who have experience living with ADHD in situations of incarceration, clinicians who have experience making treatment decisions with these patients, and facility staff who have experience with the socialization and security of PWAI within institutions. A research agenda should include consensus-based treatments; use consistent diagnostic criteria, treatment protocols and outcome measures; and include demographically representative populations of PWAI.

Our study has not captured grey literature. It has not provided quantitative synthesis of data, nor has it provided systematic assessment of reliability of the literature. However, the number of studies and participants, and the methodological diversity among these, renders more systematic synthesis inappropriate. Our scoping review approach to this question was well suited to mapping the characteristics of the knowledge base, and for qualitatively exploring the range of methods and findings.

## Conclusions

The current literature on ADHD treatments in correctional settings is diverse and contentious. Additional high quality research is needed to improve health outcomes for individuals with ADHD experiencing incarceration.

## Data Availability

All articles resulting from each step in the search and screening process have been retained by the authors and are available on request.
